# Supplementary material for: Nonequilibrium Kondo effect in a graphene-coupled quantum dot in the presence of a magnetic field
Source: Beilstein J Nanotechnol. 2020 Jan 20;11:225–39. doi: 10.3762/bjnano.11.17 (PMC7006482; doi:10.3762/bjnano.11.17)
Supplement: File 1 — Derivation of the QD retarded Green’s function, of self-energies and verification of the developed analytical method. [file Beilstein_J_Nanotechnol-11-225-s001.pdf]

BEILSTEIN JOURNAL OF NANOTECHNOLOGY

Supporting Information

for

Nonequilibrium Kondo effect in a graphene-coupled quantum dot in the presence of a magnetic field

Levente Máthé and Ioan Grosu

Beilstein J. Nanotechnol. **2020,** *11,* 225–239. doi:10.3762/bjnano.11.17

Derivation of the QD retarded Green's function, of self-energies and verification of the developed analytical method

License and Terms: This is a supporting information file under the terms of the Creative Commons Attribution License (https://creativecommons.org/licenses/by/4.0). Please note that the reuse, redistribution and reproduction in particular requires that the authors and source are credited.
The license is subject to the *Beilstein Journal of Nanotechnology* terms and conditions: (https://www.beilstein-journals.org/bjnano)

Appendix A: Green's function of the dot

The retarded Green's function is defined as $\langle\langle A(t)|B(0)\rangle\rangle_t^r = -i\theta(t)\langle\{A(t),B(0)\}\rangle$, where A and B are fermionic operators and $\theta(t)$ is the Heaviside function [1-3]. Its Fourier transform reads $\langle\langle A|B\rangle\rangle_\omega^r$. The equation of motion of the retarded Green's function in energy space is $\omega^+\langle\langle A|B\rangle\rangle_\omega^r + \langle\langle [H,A]|B\rangle\rangle_\omega^r = \langle\{A,B\}\rangle$, where $\omega^+ = \omega + i\delta$, with δ being a positive infinitesimal [1,3]. We can define the dot retarded Green's function as $G_{d\sigma}^r(\omega) = \langle\langle d_\sigma|d_\sigma^\dagger\rangle\rangle_\omega^r$ when replacing $A(B)$ by $d_\sigma(d_\sigma^\dagger)$ in the above notations. The equation of motion for $G_{d\sigma}^r(\omega)$ is:

$$(\omega^+ - \varepsilon_{d\sigma})G_{d\sigma}^r(\omega) = 1 + U\langle\langle d_\sigma n_{\bar{\sigma}}|d_\sigma^\dagger\rangle\rangle_\omega^r + \sum_{\alpha,s}\int_{-k_c}^{+k_c} dk V(k)\langle\langle c_{\alpha sk\sigma}|d_\sigma^\dagger\rangle\rangle_\omega^r. \tag{S1}$$

The equation for the term $\langle\langle c_{\alpha sk\sigma}|d_\sigma^\dagger\rangle\rangle_\omega^r$ reads:

$$\langle\langle c_{\alpha sk\sigma}|d_\sigma^\dagger\rangle\rangle_\omega^r = \frac{V(k)}{\omega^+ - \varepsilon_k}\langle\langle d_\sigma|d_\sigma^\dagger\rangle\rangle_\omega^r. \tag{S2}$$

We define the $\Sigma_0^r(\omega)$ self-energy as:

$$\Sigma_0^r(\omega) = \sum_{\alpha,s}\int_{-k_c}^{+k_c} dk\frac{V(k)^2}{\omega^+ - \varepsilon_k} = -2\eta\left(\omega\ln\left|\frac{D^2-\omega^2}{\omega^2}\right| + i\pi|\omega|\theta(D-|\omega|)\right), \tag{S3}$$

where we used the Eq. (S31) and introduced a dimensionless parameter as $\eta = 2(\tilde{V}/\hbar v_F)^2$. By substituting Eq. (S2) into Eq. (S1) with Eq. (S3) we have:

$$(\omega^+ - \varepsilon_{d\sigma} - \Sigma_0^r(\omega))G_{d\sigma}^r(\omega) = 1 + U\langle\langle d_\sigma n_{\bar{\sigma}}|d_\sigma^\dagger\rangle\rangle_\omega^r. \tag{S4}$$

The equation of motion for $\langle\langle d_\sigma n_{\bar\sigma}|d_\sigma^\dagger\rangle\rangle_\omega^r$ is:

$$(\omega^+ - \varepsilon_{d\sigma} - U)\langle\langle d_\sigma n_{\bar\sigma}|d_\sigma^\dagger\rangle\rangle_\omega^r = \langle n_{\bar\sigma}\rangle$$
$$+\sum_{\alpha,s}\int_{-k_c}^{+k_c} dk V(k)\Big[\langle\langle c_{\alpha sk\sigma} n_{\bar\sigma}|d_\sigma^\dagger\rangle\rangle_\omega^r + \langle\langle d_{\bar\sigma}^\dagger c_{\alpha sk\bar\sigma} d_\sigma|d_\sigma^\dagger\rangle\rangle_\omega^r - \langle\langle c_{\alpha sk\bar\sigma}^\dagger d_{\bar\sigma} d_\sigma|d_\sigma^\dagger\rangle\rangle_\omega^r\Big]. \tag{S5}$$

To determine the Green's function of the quantum dot, we need to calculate the new higher-order correlation functions that appear on the right-hand side of Eq. (S5). The equations of motion for these terms are expressed as:

$$\Omega_k^{(0)}(\omega)\langle\langle c_{\alpha sk\sigma} n_{\bar\sigma}|d_\sigma^\dagger\rangle\rangle_\omega^r = V(k)\langle\langle d_\sigma n_{\bar\sigma}|d_\sigma^\dagger\rangle\rangle_\omega^r$$
$$-\sum_{\alpha',s'}\int_{-k_c}^{+k_c} dk' V(k')\Big[\langle\langle c_{\alpha sk\sigma} c_{\alpha's'k'\bar\sigma}^\dagger d_{\bar\sigma}|d_\sigma^\dagger\rangle\rangle_\omega^r + \langle\langle c_{\alpha sk\sigma} c_{\alpha's'k'\bar\sigma} d_{\bar\sigma}^\dagger|d_\sigma^\dagger\rangle\rangle_\omega^r\Big], \tag{S6}$$

$$\Omega_{k\sigma}^{(1)}(\omega)\langle\langle d_{\bar\sigma}^\dagger c_{\alpha sk\bar\sigma} d_\sigma|d_\sigma^\dagger\rangle\rangle_\omega^r = \langle d_{\bar\sigma}^\dagger c_{\alpha sk\bar\sigma}\rangle + V(k)\langle\langle d_\sigma n_{\bar\sigma}|d_\sigma^\dagger\rangle\rangle_\omega^r$$
$$+\sum_{\alpha',s'}\int_{-k_c}^{+k_c} dk' V(k')\Big[\langle\langle d_{\bar\sigma}^\dagger c_{\alpha sk\bar\sigma} c_{\alpha's'k'\sigma}|d_\sigma^\dagger\rangle\rangle_\omega^r - \langle\langle c_{\alpha's'k'\bar\sigma}^\dagger c_{\alpha sk\bar\sigma} d_\sigma|d_\sigma^\dagger\rangle\rangle_\omega^r\Big], \tag{S7}$$

$$\Omega_{k\sigma}^{(2)}(\omega)\langle\langle c_{\alpha sk\bar\sigma}^\dagger d_{\bar\sigma} d_\sigma|d_\sigma^\dagger\rangle\rangle_\omega^r = \langle c_{\alpha sk\bar\sigma}^\dagger d_{\bar\sigma}\rangle - V(k)\langle\langle d_\sigma n_{\bar\sigma}|d_\sigma^\dagger\rangle\rangle_\omega^r$$
$$+\sum_{\alpha',s'}\int_{-k_c}^{+k_c} dk' V(k')\Big[\langle\langle c_{\alpha sk\bar\sigma}^\dagger c_{\alpha's'k'\bar\sigma} d_\sigma|d_\sigma^\dagger\rangle\rangle_\omega^r - \langle\langle c_{\alpha sk\bar\sigma}^\dagger c_{\alpha's'k'\sigma} d_{\bar\sigma}|d_\sigma^\dagger\rangle\rangle_\omega^r\Big], \tag{S8}$$

where the following notations are used: $\Omega_k^{(0)}(\omega) \equiv \omega^+ - \varepsilon_k$, $\Omega_{k\sigma}^{(1)}(\omega) \equiv \omega^+ - \varepsilon_k + (\varepsilon_{d\bar\sigma} - \varepsilon_{d\sigma})$ and $\Omega_{k\sigma}^{(2)}(\omega) \equiv \omega^+ + \varepsilon_k - (\varepsilon_{d\bar\sigma} + \varepsilon_{d\sigma}) - U$. To obtain an analytical formula for the Green's function of the quantum dot, we have to truncate the higher-order correlation functions that appear in Eqs. (S6)-(S8) by using an approximation method. In order to do this, we apply the broadly used Lacroix decoupling scheme [2] that leads to close the infinite number of higher-order correlation functions. By performing the approximations and substituting the resulting equations into the Eq. (S5) for the

Green's function of the quantum dot, we obtain:

$$G^r_{d\sigma}(\omega) = \frac{\Pi^{(1)}_\sigma(\omega) + U\left[\langle n_{\bar\sigma}\rangle + \Pi^{(2)}_\sigma(\omega)\right]}{\Pi^{(1)}_\sigma(\omega)\left[\omega - \varepsilon_{d\sigma} - \Sigma^r_0(\omega)\right] - U\Pi^{(3)}_\sigma(\omega)}, \tag{S9}$$

where we introduced the notations:

$$\Pi^{(1)}_\sigma(\omega) = \omega - \varepsilon_{d\sigma} - U - \Sigma^r_0(\omega) - \sum_{\alpha,s}\int_{-k_c}^{+k_c} dk V(k)^2\left[\frac{1}{\Omega^{(1)}_{k\sigma}} + \frac{1}{\Omega^{(2)}_{k\sigma}}\right], \tag{S10}$$

$$\Pi^{(2)}_\sigma(\omega) = \sum_{\alpha,s}\int_{-k_c}^{+k_c} dk V(k)\left[\frac{\langle d^\dagger_{\bar\sigma} c_{\alpha sk\bar\sigma}\rangle}{\Omega^{(1)}_{k\sigma}} - \frac{\langle c^\dagger_{\alpha sk\bar\sigma} d_{\bar\sigma}\rangle}{\Omega^{(2)}_{k\sigma}}\right], \tag{S11}$$

$$\begin{aligned}\Pi^{(3)}_\sigma(\omega) &= \Sigma^r_0(\omega)\Pi^{(2)}_\sigma(\omega)\\ &- \sum_{\substack{\alpha,s\\ \alpha',s'}}\iint_{-k_c}^{+k_c} dk dk' V(k)V(k')\left[\frac{\langle c^\dagger_{\alpha's'k'\bar\sigma} c_{\alpha sk\bar\sigma}\rangle}{\Omega^{(1)}_{k\sigma}} + \frac{\langle c^\dagger_{\alpha sk\bar\sigma} c_{\alpha's'k'\bar\sigma}\rangle}{\Omega^{(2)}_{k\sigma}}\right].\end{aligned} \tag{S12}$$

The average values of the mixing operators in the above relations are treated non-self-consistently, following the Meir approximation [4], i.e. $\langle d^\dagger_{\bar\sigma} c_{\alpha sk\bar\sigma}\rangle = \langle c^\dagger_{\alpha sk\bar\sigma} d_{\bar\sigma}\rangle \approx 0$ and $\langle c^\dagger_{\alpha's'k'\bar\sigma} c_{\alpha sk\bar\sigma}\rangle = \langle c^\dagger_{\alpha sk\bar\sigma} c_{\alpha's'k'\bar\sigma}\rangle \approx f_\alpha(\varepsilon_k)\delta_{\alpha\alpha'}\delta_{ss'}\delta(k-k')$. In this case $\Pi^{(2)}_\sigma(\omega) \approx 0$, the retarded Green's function for the quantum dot reduces:

$$\begin{aligned}G^r_{d\sigma}(\omega) &= \frac{1-\langle n_{\bar\sigma}\rangle}{\omega - \varepsilon_{d\sigma} - \Sigma^r_0(\omega) + U\frac{\Sigma_{3\sigma}(\omega)+\Sigma_{4\sigma}(\omega)}{\omega-\varepsilon_{d\sigma}-U-\Sigma^r_0(\omega)-\Sigma_{1\sigma}(\omega)-\Sigma_{2\sigma}(\omega)}}\\ &+ \frac{\langle n_{\bar\sigma}\rangle}{\omega - \varepsilon_{d\sigma} - \Sigma^r_0(\omega) - U - U\frac{\Sigma_{1\sigma}(\omega)+\Sigma_{2\sigma}(\omega)-\Sigma_{3\sigma}(\omega)-\Sigma_{4\sigma}(\omega)}{\omega-\varepsilon_{d\sigma}-\Sigma^r_0(\omega)-\Sigma_{1\sigma}(\omega)-\Sigma_{2\sigma}(\omega)}},\end{aligned} \tag{S13}$$

where we defined the following self-energies by the relations:

$$\Sigma_{i\sigma}(\omega) = \sum_{\alpha,s}\int_{-k_c}^{+k_c} dk\frac{V(k)^2}{\Omega^{(i)}_{k\sigma}(\omega)} = -2\eta\left[\omega_{i\sigma}\ln\left|\frac{D^2-\omega^2_{i\sigma}}{\omega^2_{i\sigma}}\right| + i\pi|\omega_{i\sigma}|\theta(D-|\omega_{i\sigma}|)\right], \quad i=1,2, \tag{S14}$$

with shorthand notations: $\omega_{1\sigma} = \omega - \sigma\Delta\varepsilon_d$ and $\omega_{2\sigma} = \omega - 2\varepsilon_d - U$. Therefore, we have $\Sigma_{3\sigma}(\omega) = \sum_{\alpha,s}\int_{-k_c}^{+k_c} dkV(k)^2\Omega_{k\sigma}^{(1)}(\omega)^{-1}f_\alpha(\varepsilon_k) = \sum_\alpha \Sigma_{3\sigma}^{\alpha(\gamma)}(\omega)$ and $\Sigma_{4\sigma}(\omega) = \sum_{\alpha,s}\int_{-k_c}^{+k_c} dkV(k)^2\Omega_{k\sigma}^{(2)}(\omega)^{-1}f_\alpha(\varepsilon_k) = \sum_\alpha \Sigma_{4\sigma}^{\alpha(\gamma)}(\omega)$ with solutions:

$$\Sigma_{3\sigma}^{\alpha(-)}(\omega) = \eta\left[D + \omega_{1\sigma}\ln\left|\frac{\mu_\alpha - \omega_{1\sigma} - 2T}{D + \omega_{1\sigma}}\right| - R_{3\sigma}^{\alpha}(\omega) - iJ_{3\sigma}^{\alpha}(\omega)\right], \tag{S15}$$

$$\begin{aligned}\Sigma_{3\sigma}^{\alpha(0)}(\omega) = \eta\Big\{&D - T + \omega_{1\sigma}\ln\left|\frac{\omega_{1\sigma} + 2T}{D + \omega_{1\sigma}}\right| \\ &- \frac{\omega_{1\sigma}}{2}\left(1 - \frac{\omega_{1\sigma}}{2T}\right)\ln\left|\frac{(\omega_{1\sigma} + 2T)(\omega_{1\sigma} - 2T)}{\omega_{1\sigma}^2}\right| - iJ_{3\sigma}^{\alpha}(\omega)\Big\},\end{aligned} \tag{S16}$$

$$\Sigma_{3\sigma}^{\alpha(+)}(\omega) = \eta\left[D - \omega_{1\sigma}\ln\left|\frac{(D + \omega_{1\sigma})(\mu_\alpha - \omega_{1\sigma} - 2T)}{\omega_{1\sigma}^2}\right| + R_{3\sigma}^{\alpha}(\omega) - iJ_{3\sigma}^{\alpha}(\omega)\right], \tag{S17}$$

where we introduced the following relations:

$$R_{3\sigma}^{\alpha}(\omega) = \omega_{1\sigma} - \mu_\alpha + \frac{\omega_{1\sigma}}{2}\left(1 + \frac{\mu_\alpha - \omega_{1\sigma}}{2T}\right)\ln\left|\frac{\mu_\alpha - \omega_{1\sigma} - 2T}{\mu_\alpha - \omega_{1\sigma} + 2T}\right|, \tag{S18}$$

$$J_{3\sigma}^{\alpha}(\omega) = \frac{\pi}{2}|\omega_{1\sigma}|\left[1 + \tanh\left(\frac{\mu_\alpha - \omega_{1\sigma}}{2T}\right)\right]\theta(D + \omega_{1\sigma}) \tag{S19}$$

and:

$$\Sigma_{4\sigma}^{\alpha(-)}(\omega) = -\eta\left[D + \omega_{2\sigma}\ln\left|\frac{D - \omega_{2\sigma}}{\mu_\alpha + \omega_{2\sigma} - 2T}\right| + R_{4\sigma}^{\alpha}(\omega) + iJ_{4\sigma}^{\alpha}(\omega)\right], \tag{S20}$$

$$\begin{aligned}\Sigma_{4\sigma}^{\alpha(0)}(\omega) = -\eta\Big\{&D - T + \omega_{2\sigma}\ln\left|\frac{D - \omega_{2\sigma}}{\omega_{2\sigma} - 2T}\right| \\ &+ \frac{\omega_{2\sigma}}{2}\left(1 + \frac{\omega_{2\sigma}}{2T}\right)\ln\left|\frac{(\omega_{2\sigma} - 2T)(\omega_{2\sigma} + 2T)}{\omega_{2\sigma}^2}\right| + iJ_{4\sigma}^{\alpha}(\omega)\Big\},\end{aligned} \tag{S21}$$

$$\Sigma_{4\sigma}^{\alpha(+)}(\omega) = -\eta\left[D + \omega_{2\sigma}\ln\left|\frac{(D - \omega_{2\sigma})(\mu_\alpha + \omega_{2\sigma} - 2T)}{\omega_{2\sigma}^2}\right| - R_{4\sigma}^{\alpha}(\omega) + iJ_{4\sigma}^{\alpha}(\omega)\right], \tag{S22}$$

where we have:

$$R_{4\sigma}^{\alpha}(\omega) = \omega_{2\sigma} + \mu_\alpha + \frac{\omega_{2\sigma}}{2}\left(1 + \frac{\mu_\alpha + \omega_{2\sigma}}{2T}\right)\ln\left|\frac{\mu_\alpha + \omega_{2\sigma} - 2T}{\mu_\alpha + \omega_{2\sigma} + 2T}\right|, \tag{S23}$$

$$J^{\alpha}_{4\sigma}(\omega) = \frac{\pi}{2}|\omega_{2\sigma}|\left[1+\tanh\left(\frac{\mu_\alpha+\omega_{2\sigma}}{2T}\right)\right]\theta(D-\omega_{2\sigma}), \tag{S24}$$

where $\gamma = -,0$ and $+$ correspond to the cases: $-D < \mu_\alpha \lesssim 0$, $\mu_\alpha = 0$ and $0 \lesssim \mu_\alpha < D$.

Appendix B: Derivation of the self-energies at finite temperature

In this section, we show a simple method to deduce self-energies $\Sigma_{3\sigma}(\omega)$ and $\Sigma_{4\sigma}(\omega)$ for finite temperatures. We introduce the self-energies by:

$$\Sigma_{3\sigma}(\omega) = \sum_{\alpha,s}\int_{-k_c}^{+k_c} dk \frac{V(k)^2}{\Omega^{(1)}_{k\sigma}(\omega)} f(\varepsilon_k - \mu_\alpha) = \sum_{\alpha}\Sigma^{\alpha(\gamma)}_{3\sigma}(\omega) \tag{S25}$$

and:

$$\Sigma_{4\sigma}(\omega) = \sum_{\alpha,s}\int_{-k_c}^{+k_c} dk \frac{V(k)^2}{\Omega^{(2)}_{k\sigma}(\omega)} f(\varepsilon_k - \mu_\alpha) = \sum_{\alpha}\Sigma^{\alpha(\gamma)}_{4\sigma}(\omega), \tag{S26}$$

with:

$$\Sigma^{\alpha(\gamma)}_{3\sigma}(\omega) = \eta\int_{-D}^{+D} d\varepsilon \frac{|\varepsilon| f(\varepsilon-\mu_\alpha)}{-\varepsilon+\omega_{1\sigma}+i\delta} = \eta I^{\alpha(\gamma)}_{3\sigma}(\omega) \tag{S27}$$

and:

$$\Sigma^{\alpha(\gamma)}_{4\sigma}(\omega) = \eta\int_{-D}^{+D} d\varepsilon \frac{|\varepsilon| f(\varepsilon-\mu_\alpha)}{\varepsilon+\omega_{2\sigma}+i\delta} = \eta I^{\alpha(\gamma)}_{4\sigma}(\omega), \tag{S28}$$

where we introduced the notation $\varepsilon = \hbar v_F k$. Note that $\Sigma^{\alpha(\gamma)}_{3\sigma}(\omega)$ and $\Sigma^{\alpha(\gamma)}_{4\sigma}(\omega)$ explicitly depend on ω through $\omega_{1\sigma}$ and $\omega_{2\sigma}$, respectively. Furthermore, by changing the variable $\beta(\varepsilon-\mu_\alpha) = x$ where $\beta = 1/T$, then the Fermi function $f(\varepsilon-\mu_\alpha)$ can be expressed as:

$$f(x) = \frac{1}{2}\left[1-\tanh\left(\frac{x}{2}\right)\right], \tag{S29}$$

where $\tanh(x/2)$ has the properties [5]:

$$\tanh\left(\frac{x}{2}\right) \approx \begin{cases} -1 & \text{if } x < -2 \\ \frac{x}{2} & \text{if } -2 < x < 2 \\ +1 & \text{if } x > 2. \end{cases} \tag{S30}$$

The following calculations will be based on the properties of function $\tanh(x/2)$ outlined in Eq. (S30). We also use the Dirac identity [6]:

$$\frac{1}{x \pm i\eta} = \mathscr{P}\frac{1}{x} \mp i\pi\delta(x), \tag{S31}$$

where η is a positive infinitesimal, $\mathscr{P}$ is the Cauchy principal value and $\delta(x)$ being the Dirac delta function. We implicity applied this relation to deduce $\Sigma_0^r(\omega)$ and $\Sigma_{i\sigma}(\omega)$ in Eqs. (S3) and (S14). From Eq. (S27) we write for $I_{3\sigma}^{\alpha(\gamma)}(\omega) = I_{3\sigma}^{\alpha(\gamma)1}(\omega) - I_{3\sigma}^{\alpha(\gamma)2}(\omega)$ with:

$$I_{3\sigma}^{\alpha(\gamma)1}(\omega) = \int_{-D}^{0} d\varepsilon \frac{\varepsilon \cdot f(\varepsilon - \mu_\alpha)}{\varepsilon - \omega_{1\sigma} - i\delta}, \tag{S32}$$

$$I_{3\sigma}^{\alpha(\gamma)2}(\omega) = \int_{0}^{D} d\varepsilon \frac{\varepsilon \cdot f(\varepsilon - \mu_\alpha)}{\varepsilon - \omega_{1\sigma} - i\delta}. \tag{S33}$$

Firstly, we assume that $0 \lesssim \mu_\alpha < D$ and $0 \lesssim \omega_{1\sigma} < D'$ where $D' > D$ is arbitrarily introduced. Using Eqs. (S29)-(S31) then $I_{3\sigma}^{\alpha(+)1}(\omega)$ and $I_{3\sigma}^{\alpha(+)2}(\omega)$ can be calculated as:

$$\begin{aligned} I_{3\sigma}^{\alpha(+)1}(\omega) &\approx \frac{1}{2\beta} \int_{-\beta(D+\mu_\alpha)}^{-\beta\mu_\alpha} \frac{dx(x+\beta\mu_\alpha)}{x+\beta(\mu_\alpha - \omega_{1\sigma})}\left[1 - \tanh\left(\frac{x}{2}\right)\right] \\ &\approx D + \omega_{1\sigma} \ln\left|\frac{\omega_{1\sigma}}{D+\omega_{1\sigma}}\right|, \end{aligned} \tag{S34}$$

where the imaginary part in the integral has been neglected due to the limits of integration, and:

$$I_{3\sigma}^{\alpha(+)2}(\omega) = \frac{1}{2\beta}\int\limits_{-\beta\mu_\alpha}^{\beta(D-\mu_\alpha)} \frac{dx(x+\beta\mu_\alpha)}{x+\beta(\mu_\alpha-\omega_{1\sigma})-i\eta}\left[1-\tanh\left(\frac{x}{2}\right)\right]$$
$$\approx -\omega_{1\sigma}\ln\left|\frac{\omega_{1\sigma}}{\mu_\alpha-\omega_{1\sigma}-2T}\right| - \frac{\omega_{1\sigma}}{2}\left(1+\frac{\mu_\alpha-\omega_{1\sigma}}{2T}\right)\ln\left|\frac{\mu_\alpha-\omega_{1\sigma}-2T}{\mu_\alpha-\omega_{1\sigma}+2T}\right|$$
$$+\mu_\alpha-\omega_{1\sigma}+i\frac{\pi}{2}\omega_{1\sigma}\left[1+\tanh\left(\frac{\mu_\alpha-\omega_{1\sigma}}{2T}\right)\right], \tag{S35}$$

where $\eta = \delta\beta \to 0^+$. We consider the case: $0 \lesssim \mu_\alpha < D$ and $-D' < \omega_{1\sigma} \lesssim 0$. Introducing $\omega_{1\sigma}^+ = -\omega_{1\sigma}$ in the same way we find:

$$I_{3\sigma}^{\alpha(+)1}(\omega) = \frac{1}{2\beta}\int\limits_{-\beta(D+\mu_\alpha)}^{-\beta\mu_\alpha} \frac{dx(x+\beta\mu_\alpha)}{x+\beta(\mu_\alpha+\omega_{1\sigma}^+)-i\eta}\left[1-\tanh\left(\frac{x}{2}\right)\right]$$
$$\approx D-\omega_{1\sigma}^+\ln\left|\frac{\omega_{1\sigma}^+}{D-\omega_{1\sigma}^+}\right| - i\frac{\pi}{2}\omega_{1\sigma}^+\left[1+\tanh\left(\frac{\mu_\alpha+\omega_{1\sigma}^+}{2T}\right)\right]\theta(D-\omega_{1\sigma}^+). \tag{S36}$$

Furthermore, we have:

$$I_{3\sigma}^{\alpha(+)2}(\omega) \approx \frac{1}{2\beta}\int\limits_{-\beta\mu_\alpha}^{\beta(D-\mu_\alpha)} \frac{dx(x+\beta\mu_\alpha)}{x+\beta(\mu_\alpha+\omega_{1\sigma}^+)}\left[1-\tanh\left(\frac{x}{2}\right)\right] \approx \mu_\alpha+\omega_{1\sigma}^+$$
$$+\omega_{1\sigma}^+\ln\left|\frac{\omega_{1\sigma}^+}{\mu_\alpha+\omega_{1\sigma}^+-2T}\right| - \frac{\omega_{1\sigma}^+}{2}\left(1+\frac{\mu_\alpha+\omega_{1\sigma}^+}{2T}\right)\ln\left|\frac{\mu_\alpha+\omega_{1\sigma}^++2T}{\mu_\alpha+\omega_{1\sigma}^+-2T}\right|. \tag{S37}$$

Combining Eqs. (S34)-(S37) we simply obtain $I_{3\sigma}^{\alpha(+)}$ defined for the full range of the energy, $-D' < \omega_{1\sigma} < D'$:

$$I_{3\sigma}^{\alpha(+)}(\omega) \approx D-\mu_\alpha+\omega_{1\sigma}-\omega_{1\sigma}\ln\left|\frac{(D+\omega_{1\sigma})(\mu_\alpha-\omega_{1\sigma}-2T)}{\omega_{1\sigma}^2}\right|$$
$$+\frac{\omega_{1\sigma}}{2}\left(1+\frac{\mu_\alpha-\omega_{1\sigma}}{2T}\right)\ln\left|\frac{\mu_\alpha-\omega_{1\sigma}-2T}{\mu_\alpha-\omega_{1\sigma}+2T}\right| \tag{S38}$$
$$-i\frac{\pi}{2}|\omega_{1\sigma}|\left[1+\tanh\left(\frac{\mu_\alpha-\omega_{1\sigma}}{2T}\right)\right]\theta(D+\omega_{1\sigma}).$$

Assuming that $-D < \mu_\alpha \lesssim 0$ and $0 \lesssim \omega_{1\sigma} < D'$ and introducing $\mu_\alpha^+ = -\mu_\alpha$, thus, one finds:

$$\begin{aligned} I_{3\sigma}^{\alpha(-)1}(\omega) &\approx \frac{1}{2\beta} \int\limits_{-\beta(D-\mu_\alpha^+)}^{\beta\mu_\alpha^+} \frac{dx(x-\beta\mu_\alpha^+)}{x-\beta(\mu_\alpha^+ + \omega_{1\sigma})} \left[1-\tanh\left(\frac{x}{2}\right)\right] \approx D - \mu_\alpha^+ - \omega_{1\sigma} \\ &+ \omega_{1\sigma} \ln\left|\frac{\mu_\alpha^+ + \omega_{1\sigma} + 2T}{D+\omega_{1\sigma}}\right| + \frac{\omega_{1\sigma}}{2}\left(1-\frac{\mu_\alpha^+ + \omega_{1\sigma}}{2T}\right)\ln\left|\frac{\mu_\alpha^+ + \omega_{1\sigma} - 2T}{\mu_\alpha^+ + \omega_{1\sigma} + 2T}\right|, \end{aligned} \tag{S39}$$

$$\begin{aligned} I_{3\sigma}^{\alpha(-)2}(\omega) &= \frac{1}{2\beta} \int\limits_{\beta\mu_\alpha^+}^{\beta(D+\mu_\alpha^+)} \frac{dx(x-\beta\mu_\alpha^+)}{x-\beta(\mu_\alpha^+ + \omega_{1\sigma}) - i\eta} \left[1-\tanh\left(\frac{x}{2}\right)\right] \\ &\approx i\frac{\pi}{2}\omega_{1\sigma}\left[1-\tanh\left(\frac{\mu_\alpha^+ + \omega_{1\sigma}}{2T}\right)\right]. \end{aligned} \tag{S40}$$

In the case of $-D < \mu_\alpha \lesssim 0$, $-D' < \omega_{1\sigma} \lesssim 0$ and introducing $\mu_\alpha^+ = -\mu_\alpha$ and $\omega_{1\sigma}^+ = -\omega_{1\sigma}$ we have:

$$\begin{aligned} I_{3\sigma}^{\alpha(-)1}(\omega) &= \frac{1}{2\beta} \int\limits_{-\beta(D-\mu_\alpha^+)}^{\beta\mu_\alpha^+} \frac{dx(x-\beta\mu_\alpha^+)}{x-\beta(\mu_\alpha^+ - \omega_{1\sigma}^+) - i\eta} \left[1-\tanh\left(\frac{x}{2}\right)\right] \approx D - \mu_\alpha^+ + \omega_{1\sigma}^+ \\ &- \omega_{1\sigma}^+ \ln\left|\frac{\mu_\alpha^+ - \omega_{1\sigma}^+ + 2T}{D-\omega_{1\sigma}^+}\right| - \frac{\omega_{1\sigma}^+}{2}\left(1-\frac{\mu_\alpha^+ - \omega_{1\sigma}^+}{2T}\right)\ln\left|\frac{\mu_\alpha^+ - \omega_{1\sigma}^+ - 2T}{\mu_\alpha^+ - \omega_{1\sigma}^+ + 2T}\right| \\ &- i\frac{\pi}{2}\omega_{1\sigma}^+\left[1-\tanh\left(\frac{\mu_\alpha^+ - \omega_{1\sigma}^+}{2T}\right)\right]\theta(D-\omega_{1\sigma}^+), \end{aligned} \tag{S41}$$

$$I_{3\sigma}^{\alpha(-)2}(\omega) \approx \frac{1}{2\beta} \int\limits_{\beta\mu_\alpha^+}^{\beta(D+\mu_\alpha^+)} \frac{dx(x-\beta\mu_\alpha^+)}{x-\beta(\mu_\alpha^+ - \omega_{1\sigma}^+)} \left[1-\tan\left(\frac{x}{2}\right)\right] \approx 0. \tag{S42}$$

Comparing Eqs. (S39)-(S40) with Eqs. (S41)-(S42) one finds the $I_{3\sigma}^{\alpha(-)}$ for the entire energy domain, $-D' < \omega_{1\sigma} < D'$:

$$\begin{aligned} I_{3\sigma}^{\alpha(-)}(\omega) &\approx D + \mu_\alpha - \omega_{1\sigma} + \omega_{1\sigma}\ln\left|\frac{\mu_\alpha - \omega_{1\sigma} - 2T}{D+\omega_{1\sigma}}\right| \\ &+ \frac{\omega_{1\sigma}}{2}\left(1+\frac{\mu_\alpha - \omega_{1\sigma}}{2T}\right)\ln\left|\frac{\mu_\alpha - \omega_{1\sigma} + 2T}{\mu_\alpha - \omega_{1\sigma} - 2T}\right| \\ &- i\frac{\pi}{2}|\omega_{1\sigma}|\left[1+\tanh\left(\frac{\mu_\alpha - \omega_{1\sigma}}{2T}\right)\right]\theta(D+\omega_{1\sigma}). \end{aligned} \tag{S43}$$

Now, we consider the case $\mu_\alpha = 0$ and $0 \lesssim \omega_{1\sigma} < D'$ and find:

$$I_{3\sigma}^{\alpha(0)1}(\omega) \approx \frac{1}{2\beta} \int_{-\beta D}^{0} \frac{xdx}{x - \beta\omega_{1\sigma}} \left[1 - \tanh\left(\frac{x}{2}\right)\right]$$
$$\approx D + \omega_{1\sigma} \ln\left|\frac{\omega_{1\sigma} + 2T}{D + \omega_{1\sigma}}\right| + \frac{\omega_{1\sigma}}{2}\left(1 - \frac{\omega_{1\sigma}}{2T}\right) \ln\left|\frac{\omega_{1\sigma}}{\omega_{1\sigma} + 2T}\right| - \frac{T}{2} - \frac{\omega_{1\sigma}}{2}, \tag{S44}$$

$$I_{3\sigma}^{\alpha(0)2}(\omega) = \frac{1}{2\beta} \int_{0}^{\beta D} \frac{xdx}{x - \beta\omega_{1\sigma} - i\eta} \left[1 - \tanh\left(\frac{x}{2}\right)\right] \approx \frac{T}{2} - \frac{\omega_{1\sigma}}{2}$$
$$+ \frac{\omega_{1\sigma}}{2}\left(1 - \frac{\omega_{1\sigma}}{2T}\right) \ln\left|\frac{\omega_{1\sigma} - 2T}{\omega_{1\sigma}}\right| + i\frac{\pi}{2}\omega_{1\sigma}\left[1 - \tanh\left(\frac{\omega_{1\sigma}}{2T}\right)\right]. \tag{S45}$$

For $\mu_\alpha = 0$ and $-D' < \omega_{1\sigma} \lesssim 0$ with $\omega_{1\sigma}^+ = -\omega_{1\sigma}$ we have:

$$I_{3\sigma}^{\alpha(0)1}(\omega) = \frac{1}{2\beta} \int_{-\beta D}^{0} \frac{xdx}{x + \beta\omega_{1\sigma}^+ - i\eta} \left[1 - \tanh\left(\frac{x}{2}\right)\right] \approx D - \frac{T}{2} + \frac{\omega_{1\sigma}^+}{2}$$
$$+ \omega_{1\sigma}^+ \ln\left|\frac{\omega_{1\sigma}^+ - D}{\omega_{1\sigma}^+ - 2T}\right| - \frac{\omega_{1\sigma}^+}{2}\left(1 + \frac{\omega_{1\sigma}^+}{2T}\right) \ln\left|\frac{\omega_{1\sigma}^+}{\omega_{1\sigma}^+ - 2T}\right|$$
$$- i\frac{\pi}{2}\omega_{1\sigma}^+\left[1 + \tanh\left(\frac{\omega_{1\sigma}^+}{2T}\right)\right]\theta(D - \omega_{1\sigma}^+), \tag{S46}$$

$$I_{3\sigma}^{\alpha(0)2}(\omega) \approx \frac{1}{2\beta} \int_{0}^{\beta D} \frac{xdx}{x + \beta\omega_{1\sigma}^+} \left[1 - \tanh\left(\frac{x}{2}\right)\right]$$
$$\approx \frac{T}{2} + \frac{\omega_{1\sigma}^+}{2} - \frac{\omega_{1\sigma}^+}{2}\left(1 + \frac{\omega_{1\sigma}^+}{2T}\right) \ln\left|\frac{\omega_{1\sigma}^+ + 2T}{\omega_{1\sigma}^+}\right|. \tag{S47}$$

Using Eqs. (S44)-(S47) for the full energy domain $I_{3\sigma}^{\alpha(0)}$ can be expressed as:

$$I_{3\sigma}^{\alpha(0)}(\omega) \approx D - T + \omega_{1\sigma} \ln\left|\frac{\omega_{1\sigma} + 2T}{D + \omega_{1\sigma}}\right|$$
$$- \frac{\omega_{1\sigma}}{2}\left(1 - \frac{\omega_{1\sigma}}{2T}\right) \ln\left|\frac{(\omega_{1\sigma} + 2T)(\omega_{1\sigma} - 2T)}{\omega_{1\sigma}^2}\right|$$
$$- i\frac{\pi}{2}|\omega_{1\sigma}|\left[1 - \tanh\left(\frac{\omega_{1\sigma}}{2T}\right)\right]\theta(D + \omega_{1\sigma}). \tag{S48}$$

Comparing Eqs. (S38), (S43) and (S48), Eqs. (S15)-(S19) can be introduced. In similar way, we can write $I_{4\sigma}^{\alpha(\gamma)}(\omega) = -I_{4\sigma}^{\alpha(\gamma)1}(\omega) + I_{4\sigma}^{\alpha(\gamma)2}(\omega)$ with:

$$I_{4\sigma}^{\alpha(\gamma)1}(\omega) = \int_{-D}^{0} d\varepsilon \frac{\varepsilon \cdot f(\varepsilon - \mu_\alpha)}{\varepsilon + \omega_{2\sigma} + i\delta}, \tag{S49}$$

$$I_{4\sigma}^{\alpha(\gamma)2}(\omega) = \int_{0}^{D} d\varepsilon \frac{\varepsilon \cdot f(\varepsilon - \mu_\alpha)}{\varepsilon + \omega_{2\sigma} + i\delta}. \tag{S50}$$

We assume that $0 \lesssim \mu_\alpha < D$ and $0 \lesssim \omega_{2\sigma} < D'$, one finds:

$$\begin{aligned} I_{4\sigma}^{\alpha(+)1}(\omega) &= \frac{1}{2\beta} \int_{-\beta(D+\mu_\alpha)}^{-\beta\mu_\alpha} \frac{dx(x+\beta\mu_\alpha)}{x+\beta(\mu_\alpha+\omega_{2\sigma})+i\eta}\left[1-\tanh\left(\frac{x}{2}\right)\right] \\ &\approx D - \omega_{2\sigma}\ln\left|\frac{\omega_{2\sigma}}{D-\omega_{2\sigma}}\right| + i\frac{\pi}{2}\omega_{2\sigma}\left[1+\tanh\left(\frac{\mu_\alpha+\omega_{2\sigma}}{2T}\right)\right]\theta(D-\omega_{2\sigma}), \end{aligned} \tag{S51}$$

$$\begin{aligned} I_{4\sigma}^{\alpha(+)2}(\omega) &\approx \frac{1}{2\beta} \int_{-\beta\mu_\alpha}^{\beta(D-\mu_\alpha)} \frac{dx(x+\beta\mu_\alpha)}{x+\beta(\mu_\alpha+\omega_{2\sigma})}\left[1-\tanh\left(\frac{x}{2}\right)\right] \approx \mu_\alpha + \omega_{2\sigma} \\ &+ \omega_{2\sigma}\ln\left|\frac{\omega_{2\sigma}}{\mu_\alpha+\omega_{2\sigma}-2T}\right| + \frac{\omega_{2\sigma}}{2}\left(1+\frac{\mu_\alpha+\omega_{2\sigma}}{2T}\right)\ln\left|\frac{\mu_\alpha+\omega_{2\sigma}-2T}{\mu_\alpha+\omega_{2\sigma}+2T}\right|. \end{aligned} \tag{S52}$$

We consider the case $0 \lesssim \mu_\alpha < D$ and $-D' < \omega_{2\sigma} \lesssim 0$, introducing $\omega_{2\sigma}^{+} = -\omega_{2\sigma}$, in the same way we have:

$$\begin{aligned} I_{4\sigma}^{\alpha(+)1}(\omega) &\approx \frac{1}{2\beta} \int_{-\beta(D+\mu_\alpha)}^{-\beta\mu_\alpha} \frac{dx(x+\beta\mu_\alpha)}{x+\beta(\mu_\alpha-\omega_{2\sigma}^{+})}\left[1-\tanh\left(\frac{x}{2}\right)\right] \\ &\approx D + \omega_{2\sigma}^{+}\ln\left|\frac{\omega_{2\sigma}^{+}}{D+\omega_{2\sigma}^{+}}\right|, \end{aligned} \tag{S53}$$

$$I_{4\sigma}^{\alpha(+)2}(\omega) = \frac{1}{2\beta}\int\limits_{-\beta\mu_\alpha}^{\beta(D-\mu_\alpha)} \frac{dx(x+\beta\mu_\alpha)}{x+\beta(\mu_\alpha-\omega_{2\sigma}^{+})+i\eta}\left[1-\tanh\left(\frac{x}{2}\right)\right] \approx \mu_\alpha - \omega_{2\sigma}^{+}$$
$$+\,\omega_{2\sigma}^{+}\ln\left|\frac{\mu_\alpha-\omega_{2\sigma}^{+}-2T}{\omega_{2\sigma}^{+}}\right| + \frac{\omega_{2\sigma}^{+}}{2}\left(1+\frac{\mu_\alpha-\omega_{2\sigma}^{+}}{2T}\right)\ln\left|\frac{\mu_\alpha-\omega_{2\sigma}^{+}+2T}{\mu_\alpha-\omega_{2\sigma}^{+}-2T}\right| \tag{S54}$$
$$-\,i\frac{\pi}{2}\omega_{2\sigma}^{+}\left[1+\tanh\left(\frac{\mu_\alpha-\omega_{2\sigma}^{+}}{2T}\right)\right].$$

Combining Eqs. (S51)-(S54), we can express the $I_{4\sigma}^{\alpha(+)}$ defined for the entire range of energy, $-D' < \omega_{2\sigma} < D'$:

$$I_{4\sigma}^{\alpha(+)}(\omega) \approx -D+\mu_\alpha+\omega_{2\sigma}+\omega_{2\sigma}\ln\left|\frac{\omega_{2\sigma}^{2}}{(D-\omega_{2\sigma})(\mu_\alpha+\omega_{2\sigma}-2T)}\right|$$
$$+\,\frac{\omega_{2\sigma}}{2}\left(1+\frac{\mu_\alpha+\omega_{2\sigma}}{2T}\right)\ln\left|\frac{\mu_\alpha+\omega_{2\sigma}-2T}{\mu_\alpha+\omega_{2\sigma}+2T}\right| \tag{S55}$$
$$-\,i\frac{\pi}{2}|\omega_{2\sigma}|\left[1+\tanh\left(\frac{\mu_\alpha+\omega_{2\sigma}}{2T}\right)\right]\theta(D-\omega_{2\sigma}).$$

For $-D < \mu_\alpha \lesssim 0$ and $0 \lesssim \omega_{2\sigma} < D'$ with $\mu_\alpha^{+} = -\mu_\alpha$ we find:

$$I_{4\sigma}^{\alpha(-)1}(\omega) = \frac{1}{2\beta}\int\limits_{-\beta(D-\mu_\alpha^{+})}^{\beta\mu_\alpha^{+}} \frac{dx(x-\beta\mu_\alpha^{+})}{x-\beta(\mu_\alpha^{+}-\omega_{2\sigma})+i\eta}\left[1-\tanh\left(\frac{x}{2}\right)\right] \approx D-\mu_\alpha^{+}+\omega_{2\sigma}$$
$$-\,\omega_{2\sigma}\ln\left|\frac{\mu_\alpha^{+}-\omega_{2\sigma}+2T}{D-\omega_{2\sigma}}\right| - \frac{\omega_{2\sigma}}{2}\left(1-\frac{\mu_\alpha^{+}-\omega_{2\sigma}}{2T}\right)\ln\left|\frac{\mu_\alpha^{+}-\omega_{2\sigma}-2T}{\mu_\alpha^{+}-\omega_{2\sigma}+2T}\right| \tag{S56}$$
$$+\,i\frac{\pi}{2}\omega_{2\sigma}\left[1-\tanh\left(\frac{\mu_\alpha^{+}-\omega_{2\sigma}}{2T}\right)\right]\theta(D-\omega_{2\sigma}),$$

$$I_{4\sigma}^{\alpha(-)2}(\omega) \approx \frac{1}{2\beta}\int\limits_{\beta\mu_\alpha^{+}}^{\beta(D+\mu_\alpha^{+})} \frac{dx(x-\beta\mu_\alpha^{+})}{x-\beta(\mu_\alpha^{+}-\omega_{2\sigma})}\left[1-\tanh\left(\frac{x}{2}\right)\right] \approx 0. \tag{S57}$$

In the case of $-D < \mu_\alpha \lesssim 0$ and $-D' < \omega_{2\sigma} \lesssim 0$ we have:

$$I_{4\sigma}^{\alpha(-)1}(\omega) \approx \frac{1}{2\beta}\int\limits_{-\beta(D-\mu_\alpha^{+})}^{\beta\mu_\alpha^{+}} \frac{dx(x-\beta\mu_\alpha^{+})}{x-\beta(\mu_\alpha^{+}+\omega_{2\sigma}^{+})}\left[1-\tanh\left(\frac{x}{2}\right)\right] \approx D-\mu_\alpha^{+}-\omega_{2\sigma}^{+}$$
$$+\,\omega_{2\sigma}^{+}\ln\left|\frac{\mu_\alpha^{+}+\omega_{2\sigma}^{+}+2T}{D+\omega_{2\sigma}^{+}}\right| + \frac{\omega_{2\sigma}^{+}}{2}\left(1-\frac{\mu_\alpha^{+}+\omega_{2\sigma}^{+}}{2T}\right)\ln\left|\frac{\mu_\alpha^{+}+\omega_{2\sigma}^{+}-2T}{\mu_\alpha^{+}+\omega_{2\sigma}^{+}+2T}\right|, \tag{S58}$$

$$I_{4\sigma}^{\alpha(-)2}(\omega) = \frac{1}{2\beta}\int_{\beta\mu_\alpha^+}^{\beta(D+\mu_\alpha^+)} \frac{dx(x-\beta\mu_\alpha^+)}{x-\beta(\mu_\alpha^+ + \omega_{2\sigma}^+)+i\eta}\left[1-\tan\left(\frac{x}{2}\right)\right]$$
$$\approx -i\frac{\pi}{2}\omega_{2\sigma}^+\left[1-\tanh\left(\frac{\mu_\alpha^+ + \omega_{2\sigma}^+}{2T}\right)\right]. \tag{S59}$$

Using Eqs. (S56)-(S59), we obtain $I_{4\sigma}^{\alpha(-)}$ for $-D' < \omega_{2\sigma} < D'$:

$$I_{4\sigma}^{\alpha(-)}(\omega) \approx -D-\mu_\alpha-\omega_{2\sigma}+\omega_{2\sigma}\ln\left|\frac{\mu_\alpha+\omega_{2\sigma}-2T}{D-\omega_{2\sigma}}\right|$$
$$+\frac{\omega_{2\sigma}}{2}\left(1+\frac{\mu_\alpha+\omega_{2\sigma}}{2T}\right)\ln\left|\frac{\mu_\alpha+\omega_{2\sigma}+2T}{\mu_\alpha+\omega_{2\sigma}-2T}\right| \tag{S60}$$
$$-i\frac{\pi}{2}|\omega_{2\sigma}|\left[1+\tanh\left(\frac{\mu_\alpha+\omega_{2\sigma}}{2T}\right)\right]\theta(D-\omega_{2\sigma}).$$

We now take the case $\mu_\alpha = 0$ and $0 \lesssim \omega_{2\sigma} < D'$ and find:

$$I_{4\sigma}^{\alpha(0)1}(\omega) = \frac{1}{2\beta}\int_{-\beta D}^{0} \frac{xdx}{x+\beta\omega_{2\sigma}+i\eta}\left[1-\tanh\left(\frac{x}{2}\right)\right] \approx D-\frac{T}{2}+\frac{\omega_{2\sigma}}{2}$$
$$-\omega_{2\sigma}\ln\left|\frac{\omega_{2\sigma}-2T}{D-\omega_{2\sigma}}\right|-\frac{\omega_{2\sigma}}{2}\left(1+\frac{\omega_{2\sigma}}{2T}\right)\ln\left|\frac{\omega_{2\sigma}}{\omega_{2\sigma}-2T}\right| \tag{S61}$$
$$+i\frac{\pi}{2}\omega_{2\sigma}\left[1+\tanh\left(\frac{\omega_{2\sigma}}{2T}\right)\right]\theta(D-\omega_{2\sigma}),$$

$$I_{4\sigma}^{\alpha(0)2}(\omega) \approx \frac{1}{2\beta}\int_{0}^{\beta D} \frac{xdx}{x+\beta\omega_{2\sigma}}\left[1-\tanh\left(\frac{x}{2}\right)\right]$$
$$\approx \frac{T}{2}+\frac{\omega_{2\sigma}}{2}-\frac{\omega_{2\sigma}}{2}\left(1+\frac{\omega_{2\sigma}}{2T}\right)\ln\left|\frac{\omega_{2\sigma}+2T}{\omega_{2\sigma}}\right|. \tag{S62}$$

For $\mu_\alpha = 0$ and $-D' < \omega_{2\sigma} \lesssim 0$ with $\omega_{2\sigma}^+ = -\omega_{2\sigma}$, we obtain:

$$I_{4\sigma}^{\alpha(0)1}(\omega) \approx \frac{1}{2\beta}\int_{-\beta D}^{0} \frac{xdx}{x-\beta\omega_{2\sigma}^+}\left[1-\tanh\left(\frac{x}{2}\right)\right] \approx D-\frac{T}{2}-\frac{\omega_{2\sigma}^+}{2}$$
$$+\frac{\omega_{2\sigma}^+}{2}\left(1-\frac{\omega_{2\sigma}^+}{2T}\right)\ln\left|\frac{\omega_{2\sigma}^+}{\omega_{2\sigma}^+ + 2T}\right|+\omega_{2\sigma}^+\ln\left|\frac{\omega_{2\sigma}^+ + 2T}{D+\omega_{2\sigma}^+}\right|, \tag{S63}$$

$$I_{4\sigma}^{\alpha(0)2}(\omega) = \frac{1}{2\beta}\int_0^{\beta D}\frac{xdx}{x-\beta\omega_{2\sigma}^{+}+i\eta}\Big[1-\tanh\Big(\frac{x}{2}\Big)\Big] \approx \frac{T}{2}-\frac{\omega_{2\sigma}^{+}}{2}$$
$$+\frac{\omega_{2\sigma}^{+}}{2}\Big(1-\frac{\omega_{2\sigma}^{+}}{2T}\Big)\ln\left|\frac{\omega_{2\sigma}^{+}-2T}{\omega_{2\sigma}^{+}}\right| - i\frac{\pi}{2}\omega_{2\sigma}^{+}\Big[1-\tanh\Big(\frac{\omega_{2\sigma}^{+}}{2T}\Big)\Big]. \tag{S64}$$

Comparing Eqs. (S61)-(S62) with Eqs. (S63)-(S64), one finds the $I_{4\sigma}^{\alpha(0)}$ for the full energy domain:

$$I_{4\sigma}^{\alpha(0)}(\omega) \approx -D+T+\omega_{2\sigma}\ln\left|\frac{\omega_{2\sigma}-2T}{D-\omega_{2\sigma}}\right|$$
$$+\frac{\omega_{2\sigma}}{2}\Big(1+\frac{\omega_{2\sigma}}{2T}\Big)\ln\left|\frac{\omega_{2\sigma}^2}{(\omega_{2\sigma}-2T)(\omega_{2\sigma}+2T)}\right| \tag{S65}$$
$$-i\frac{\pi}{2}|\omega_{2\sigma}|\Big[1+\tanh\Big(\frac{\omega_{2\sigma}}{2T}\Big)\Big]\theta(D-\omega_{2\sigma}).$$

In the same way, comparing Eqs. (S55), (S60) and (S65), Eqs. (S20)-(S24) can be introduced. Note that these results are valid as well at low temperatures. For absolute zero temperature we can substitute $f_\alpha(\varepsilon_k)$ with the Heaviside function, i.e., $f_\alpha(\varepsilon_k) = \theta(\mu_\alpha-\varepsilon_k)$, and using the method presented above the integrals can be calculated.

Appendix C: The verification of an analytical solution

In this section, we compare our analytical results for $\Sigma_{3\sigma}(\omega)$ presented in Appendix B with those of Z.-G. Zhu and J. Berakdar in Ref. [7]. In order to do this, we introduce the following integral:

$$I(\omega) = \int_{-D}^{D} d\varepsilon \frac{|\varepsilon| f(\varepsilon-\mu)}{-\varepsilon+\omega+i\delta}. \tag{S66}$$

Z.-G. Zhu and J. Berakdar applied a contour integral method in complex plane and found that:

$$I(\omega) = \frac{|D|}{2}\ln\left(\frac{|D^2-\omega^2|}{(2\pi T)^2}\right) - |\mu|\psi(z) - \frac{1}{2}\left[\omega\ln\frac{|D^2-\omega^2|}{\omega^2}+i\pi|\omega|\theta(D-|\omega|)\right], \tag{S67}$$

where $z=\frac{1}{2}+\frac{\omega-\mu}{2\pi i T}$ and $\psi(z)$ is the digamma function. It can be shown that the integrating function in Eq. (S66) is not a holomorphic function, and thus the contour integral method can not be applied

for $I(\omega)$. As we shall see, their results differ from those obtained by numerical calculations (see Figure S1). Our analytical results, given by $I_{3\sigma}^{\alpha(+)}(\omega)$ in Apendix B, are in better agreement with the numerical calculations. Therefore, it can be verified that the relation (S67) does not accurately reproduce the case of absolute zero temperature.

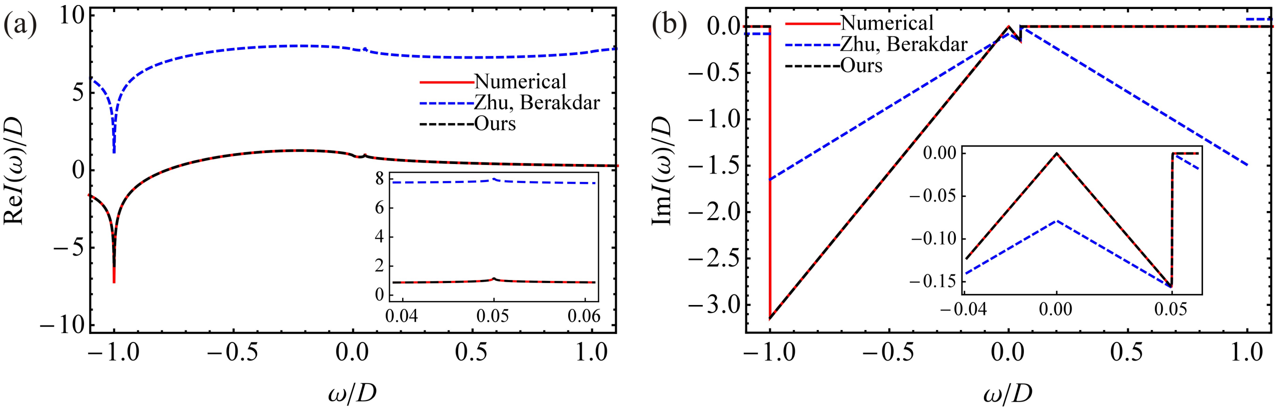

Figure S1: (a) Real part of $I(\omega)$ as a function of energy. (b) Imaginary part of $I(\omega)$ as a function of energy. The chemical potential is fixed at $\mu/D = 0.05$ and the temperature is set to be $T/D = 5 \cdot 10^{-5}$. It can be observed that our analytical results show a good agreement with the numerical calculations. The results of Z.-G. Zhu and J. Berakdar present a departure from the numerical calculations due to the mathematical method used by them.

References

1. Lim, J. S.; López, R.; Limot, L.; Simon, P. *Phys. Rev. B* **2013**, *88*, 165403. doi:10.1103/PhysRevB.88.165403.

2. Lacroix, C. *J. Phys. F: Met. Phys.* **1981**, *11*, 2389–2397. doi:10.1088/0305-4608/11/11/020.

3. Kashcheyevs, V.; Aharony, A.; Entin-Wohlman, O. *Phys. Rev. B* **2006**, *73*, 125338. doi:10.1103/PhysRevB.73.125338.

4. Meir, Y.; Wingreen, N. S.; Lee, P. A. *Phys. Rev. Lett.* **1991**, *66*, 3048–3051. doi:10.1103/PhysRevLett.66.3048.

5. Gradshteyn, I. S.; Ryzhik, I. M. *Table of integrals, series, and products*, Seventh ed.; Academic Press: Cambridge, Massachusetts, USA, 2007; p 42.

6. Nolting, W. *Fundamentals of Many-body Physics: Principles and Methods*; Springer-Verlag: Berlin, Germany, 2009; p 140.

7. Zhu, Z.-G.; Berakdar, J. *Phys. Rev. B* **2011**, *84*, 165105. doi:10.1103/PhysRevB.84.165105.
